# A Model Wife

**Published:** 1872-02

**Authors:** 


### A MODEL WIFE,

Over threescore, as fresh as a rose, and comely to behold, she goes about the house with a bunch of keys at her side, and whether in the parlor, or garden, or superintending the farm-hands, or entertaining visitors, the sons and daughters of kings and princes and the nobles of the earth, her knitting-needles are always going, and the feet of her husband (the ruling intellect of the globe), the curtains at her windows, the coverings of her beds, and the mats on her table, are evidences of her handicraft. Let our subscribers turn to the first article in the October number, and see if some of their daughters may not, in the light of it, vie with Bismarck's wife in matronly duties.

